# Thermoelectric Generator Using Polyaniline-Coated Sb_2_Se_3_/β-Cu_2_Se Flexible Thermoelectric Films

**DOI:** 10.3390/polym13091518

**Published:** 2021-05-09

**Authors:** Minsu Kim, Dabin Park, Jooheon Kim

**Affiliations:** 1 School of Chemical Engineering & Materials Science, Chung-Ang University, Seoul 06974, Korea; alstn275@gmail.com (M.K.); dragoo@naver.com (D.P.); 2 Department of Advanced Materials Engineering, Chung-Ang University, Anseong-si, Seoul 17546, Korea

**Keywords:** antimony selenide, copper selenide, polyaniline, thermoelectric generator

## Abstract

Herein, Sb_2_Se_3_ and β-Cu_2_Se nanowires are synthesized via hydrothermal reaction and water evaporation-induced self-assembly methods, respectively. The successful syntheses and morphologies of the Sb_2_Se_3_ and β-Cu_2_Se nanowires are confirmed via X-ray powder diffraction (XRD), X-ray photoelectron spectroscopy (XPS), Raman spectroscopy, field emission scanning electron microscopy (FE-SEM), and field emission transmission electron microscopy (FE-TEM). Sb_2_Se_3_ materials have low electrical conductivity which limits application to the thermoelectric generator. To improve the electrical conductivity of the Sb_2_Se_3_ and β-Cu_2_Se nanowires, polyaniline (PANI) is coated onto the surface and confirmed via Fourier-transform infrared spectroscopy (FT-IR), FE-TEM, and XPS analysis. After coating PANI, the electrical conductivities of Sb_2_Se_3_/β-Cu_2_Se/PANI composites were increased. The thermoelectric performance of the flexible Sb_2_Se_3_/β-Cu_2_Se/PANI films is then measured, and the 70%-Sb_2_Se_3_/30%-β-Cu_2_Se/PANI film is shown to provide the highest power factor of 181.61 μW/m·K^2^ at 473 K. In addition, a thermoelectric generator consisting of five legs of the 70%-Sb_2_Se_3_/30%-β-Cu_2_Se/PANI film is constructed and shown to provide an open-circuit voltage of 7.9 mV and an output power of 80.1 nW at ΔT = 30 K. This study demonstrates that the combination of inorganic thermoelectric materials and flexible polymers can generate power in wearable or portable devices.

## 1. Introduction

In recent years, thermoelectric materials have been studied for use in the thermoelectric generator (TEG) or Peltier cooler. In particular, inorganic thermoelectric materials based on Bi_2_Te_3_ [1,2], PbTe [3,4], SnSe [5,6], Cu_2_Se [7,8], skutterudites [9,10], and Zintl phases [11,12] have been studied during the past few decades. Although such inorganic thermoelectric materials exhibit better performance than their organic counterparts, they are difficult to use in wearable or portable devices due to their rigid (inflexible), brittle, heavy, costly, and toxic properties. Conversely, organic thermoelectric materials such as the conducting polymers PEDOT:PSS [13,14,15], polyaniline (PANI) [16,17,18], polythiophene [19], and polypyrrole [20,21] can exhibit lightweight, low-cost, non-toxic, and flexible properties but display low efficiency compared to their inorganic counterparts. To overcome these difficulties associated with using inorganic or organic thermoelectric materials alone, hybrid inorganic/organic thermoelectric materials have been studied in the most recent decades. In addition, the electrical conductivities of various hybrid materials have been further improved by various coating methods. For example, C. Meng et al. reported a promising improvement in the thermoelectric performance of carbon nanotubes up to 4–5 times by enwrapping the base material in PANI to provide a size-dependent energy-filtering effect [17]. In addition, D. Park et al. reported enhanced thermoelectric properties using Ag_2_Se nanowire/Polyvinylidene fluoride composite film via a solution mixing method. These studies show a combination of inorganic thermoelectric materials and polymers can be used for improvement thermoelectric performances [22].

Antimony selenide (Sb_2_Se_3_) is a chalcogenide material that is easy to synthesize in various structures such as thin films [23], nanosheets [24], and nanorods/wires [25,26]. Although Sb_2_Se_3_ has a large Seebeck coefficient of 750 μV/K, the extremely low electrical conductivity of 10^−4^ S/m [26] is a limitation in thermoelectric applications. To address this problem, an alloy of Sb_2_Se_3_ with copper selenide (Cu_2_Se) is proposed herein. Also a chalcogenide, Cu_2_Se complements Sb_2_Se_3_ by exhibiting a high electrical conductivity along with a low Seebeck coefficient. Similarly to Sb_2_Se_3_, Cu_2_Se is easy to synthesize in various structures, including films [13], nanoplates [27], and nanowires [28]. In our previous work, β-Cu_2_Se nanowires were synthesized and combined with Sb_2_Se_3_ nanowires to make the rigid disk shape composite to improve thermoelectric performance [29]. In addition to our previous work, to further improve the electrical conductivity of the Sb_2_Se_3_/β-Cu_2_Se composite, the conducting polymer polyaniline (PANI) (with an electrical conductivity of 360 S/cm [16]) was used to coat the composite surface. Moreover, in our previous work, it was found that the rigid and brittle nature of the resulting inorganic thermoelectric composites makes them difficult to use in preparing flexible films. To address this problem, a flexible thin film of polyvinylidene fluoride (PVDF) is developed. The flexible thin film with 70% β-Cu_2_Se and 30% Sb_2_Se_3_ nanowires is shown to provide a power factor of 181.61 μW/m•K^2^. This film is then used to fabricate a thermoelectric device with an output voltage of 7.9 mV and an output power of 80.1 nW at a temperature difference of 30 K. These results demonstrate that the Sb_2_Se_3_/β-Cu_2_Se/PANI flexible thin film can be used as a TEG for flexible devices.

## 2. Experimental Section

### 2.1. Materials

Sodium selenite (Na_2_SeO_3_, 99.9%), selenium powder (Se, 99.5%), and sodium dodecylbenzene sulfonate (CH_3_(CH_2_)_11_C_6_H_4_SO_3_Na, SDBS) were purchased from Sigma-Aldrich. Potassium antimony tartrate (C_8_H_4_K_2_O_12_Sb_2_·3H_2_O, 99.5) was acquired from FUJIFILM Wako Pure Chemical Corporation (Osaka, Japan). Ammonium peroxydisulfate ((NH_4_)_2_S_2_O_8_, APS, 98%) was purchased from Alfa Aesar. Sodium hydroxide (NaOH, 97%), copper(II) nitrate trihydrate (Cu(NO_3_)_2_·3H_2_O, 99%), hydrazine monohydrate (N_2_H_4_·H_2_O, 80%), tetrahydrofuran ((CH_2_)_4_O, 99%), m-cresol (C_7_H_8_O, 98%), D(+)-10-camphorsulfonic acid (C_10_H_16_O_4_S, CSA, 98%), Dimethylformamide (C_3_H_7_NO, DMF), and aniline (C_6_H_5_NH_2_, 99%) were acquired from Daejung Chemical and Metals Co., Ltd. (Seoul, Korea). All reagents were used as received without further purification.

### 2.2. Sample Preparation

#### 2.2.1. Synthesis of Sb_2_Se_3_ Nanowires

The selenium and antimony precursors were reduced using hydrazine monohydrate and synthesized to Sb_2_Se_3_ nanowires using a previously reported method [24]. In detail, potassium antimony tartrate (0.605 g) and sodium selenite (0.51 g) were completely dissolved in distilled water (100 mL) with magnetic stirring. Hydrazine monohydrate (30 mL) was then added, and the mixture was transferred to a Teflon-lined autoclave with tetrahydrofuran (40 mL). The sealed autoclave was heated to 135 °C for 9 h, then the product was centrifuged at 10,000 rpm for 1 h, washed several times with distilled water and ethanol, then dried overnight in a vacuum oven at 70 °C.

#### 2.2.2. Synthesis of β-Cu_2_Se Nanowires

The β-Cu_2_Se nanowires were synthesized via a previously reported method [28]. In detail, a mixture of Se powder (0.45 g) and NaOH (15 g) in distilled water (60 mL) was heated at 90 °C to completely dissolve the Se powder. Then, a 0.5 M Cu(NO_3_)_2_ solution (5 mL) was added, and the mixture was heated to dryness in an oven at 140 ℃ for 12 h. The precipitated product was then collected using hot distilled water, washed several times with hot distilled water and ethanol, then dried overnight in a vacuum oven at 60 ℃.

#### 2.2.3. Synthesis of Sb_2_Se_3_/β-Cu_2_Se/PANI Composite Films and Fabrication of a TEG Device

The SDBS (0.06 g) was dissolved in 1 M HCl solution (10 mL), followed by sonication for 30 min to prepare a homogeneous solution. Using an ice bath to maintain a temperature of 273 K, the aniline monomer (0.02 g) was then added to the solution with steady stirring for 12 h. Then, APS (0.04 g) was dissolved in HCl solution (5 mL) and slowly added to the prepared solution. The product was then washed three times with distilled water and dried overnight in a vacuum oven at 60 ℃ to obtain the polyaniline powder.

Using the same polymerization procedure, Sb_2_Se_3_/β-Cu_2_Se/PANI powders were synthesized by adding various ratios of Sb_2_Se_3_ and β-Cu_2_Se to 1 M HCl solution (10 mL), SDBS (0.06 g), and aniline monomer (0.02 g). The obtained Sb_2_Se_3_/β-Cu_2_Se/PANI powders were then added to 1 M ammonia solution (10 mL) and magnetically stirred for 24 h to prepare the emeraldine base PANI product. The products were then washed several times with distilled water and dried overnight in a vacuum oven at 60 ℃. To improve the electrical conductivity of the emeraldine base PANI, CSA was used as a dopant in m-cresol (10 mL) in a mole ratio of 1:2 with stirring for 24 h. The obtained powder was dried overnight in a vacuum oven at 60 ℃.

To synthesize the Sb_2_Se_3_/β-Cu_2_Se/PANI film, the Sb_2_Se_3_/β-Cu_2_Se/PANI powder (0.05 g) and polyvinylidene fluoride (PVDF) (0.025 g) were added to DMF solution (1 mL) at a weight ratio of 2:1 and sonicated for 1h to generate a homogenous mixture. The mixture was then drop-casted onto a glass substrate (18 mm×18 mm) and dried at 60 ℃ for 24 h.

To fabricate a TEG device, the Sb_2_Se_3_/β-Cu_2_Se/PANI film was cut into five (18 mm x 6 mm) strips with a thickness of 100 μm. These were then pasted onto a polyimide film and connected with copper wire. Silver (Ag) adhesive (ELCOAT P-100, CANS) was used to connect the copper wire and film.

### 2.3. Characterization

The crystalline structures of the prepared nanowire powders and de-doped PANI were examined by X-ray diffraction (XRD; D8 Advance, AXS Bruker, Billerica, US) under Cu K_α_ radiation (λ = 0.154056 nm) at 40 kV and 40 mA over a 2θ range of 10−80° at a scan rate of 1° s^−1^. The binding energies of the synthesized nanowires and Sb_2_Se_3_/β-Cu_2_Se/PANI powders were determined via X-ray photoelectron spectroscopy (XPS; K-Alpha, Thermo Fisher Scientific, Waltham, USA) using a 1486.6 eV Al K_α_ X-ray source. Fourier-transform infrared (FT-IR) spectroscopy (PerkinElmer Spectrum One) was conducted to confirm the synthesis of PANI. Raman spectra were recorded using a Raman spectrometer II (DXR2xi, Thermo Fisher Scientific, Waltham, USA) with a near infrared laser operating at 532 nm and a CCD detector. Field emission scanning electron microscopy (FE-SEM; SIGMA, Oberkochen, Germany), and field emission transmission electron microscopy (FE-TEM; JEM-F200) were used to visualize the shape and microstructures of the Sb_2_Se_3_ and Cu_2_Se nanowire samples. Energy-dispersive X-ray spectroscopy (EDS) was used to obtain the elemental mappings of the nanowire powders (JEM-F200, JEOL Ltd., Akishima, Japan). The thermoelectric properties, Seebeck coefficients, and electrical conductivities were measured in the direction parallel to the pressing direction. A four-probe method involving a homemade device with a pair of thermocouples and a pair of voltmeters was used to quantify the electrical conductivity (*σ*) between room temperature (RT) and 473 K, and the Seebeck coefficient was calculated from the relationship in Equation (1):

*S* = Δ*V*/Δ*T* (1)

where Δ*V* is the change in the thermal electromotive force and Δ*T* is the temperature difference.

In addition, the power factor (*PF*) was calculated using Equation (2):

(2) $$PF=S^{2}\cdot \sigma$$

The properties of the generator were measured using a homemade device with thermocouples and a multimeter (SENIT, A830L).

## 3. Results and Discussion

### 3.1. Crystallin Structure and Morphology of Sb_2_Se_3_ Nanowires

**Figure 1:**
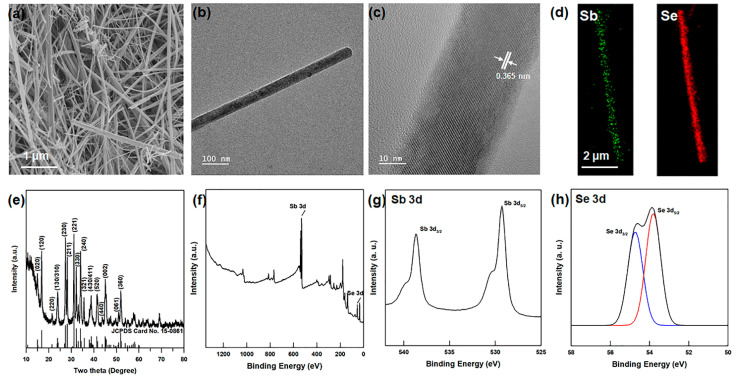
(**a**) FE-SEM image (**b**) FE-TEM (**c**) high-resolution of FE-TEM images of Sb_2_Se_3_ nanowires. EDS elemental mapping of (**d**) Sb and Se in Sb_2_Se_3_ nanowires. (**e**) XRD patterns and (**f**) XPS survey spectrum of Sb_2_Se_3_ nanowires. High-resolution XPS spectra of (**g**) Sb 3d peaks and (**h**) Se 3d peaks in Sb_2_Se_3_ nanowires.

The Sb_2_Se_3_ nanowires with diameters of 100−200 nm and lengths of 1−2 μm were successfully synthesized via the hydrothermal reaction, as shown in Figure 1a.

In addition, the FE-TEM images of the Sb_2_Se_3_ nanowires are presented in Figure 1b,c. Here, the lattice fringes of the Sb_2_Se_3_ nanowires are 0.365 nm in size, which corresponds to the (1 3 0) crystal plane [30]. The EDS mappings of the Sb_2_Se_3_ nanowires in Figure 1d,e indicate a stoichiometric atomic ratio of Sb:Se = 42.01:57.99. Moreover, the XRD pattern of the Sb_2_Se_3_ nanowires in Figure 1f reveals a diffraction peak with lattice constants of a = 1.168 nm, b = 1.172 nm, and c = 0.397 nm, corresponding to the orthorhombic structure (JCPDS #15-0681, a = 1.1633 nm, b = 1.1780 nm, and c = 0.3985 nm). The absence of any second phase peaks demonstrates the high purity of the nanowires, and the strong intensities of the (h k 0) planes indicate that the Sb_2_Se_3_ particles have a 1-dimensional nanowire structure. Further, the XPS spectra of the Sb_2_Se_3_ nanowires are presented in Figure 1g−i. Here, the XPS wide scan spectra exhibit the Sb 3d and Se 3d peaks with no O 1s peak, thus indicating that the Sb_2_O_3_ phase was not produced during the synthesis, in agreement with the above interpretation of the XRD pattern. Further, the high-resolution Sb 3d spectrum in Figure 1h exhibits the Sb 3d_5/2_ and Sb 3d_3/2_ peaks at binding energies of 529.5 and 538.8 eV, respectively, in agreement with the previously reported data [24]. Similarly, the binding energies of Se 3d_5/2_ and Se 3d_3/2_ are located at 53.91 and 54.73 eV, which is in close agreement with the previously reported data [25]. The atomic ratio of Sb:Se obtained from the XPS spectra is 41.21:58.79, which is close to that obtained from the EDS mapping data and to the stoichiometric ratio. The Raman spectrum of the Sb_2_Se_3_ nanowires is provided in Figure S1 of the Supplementary Information. Here, the peaks located at 118, 188, and 208 cm^−1^ are consistent with the Sb_2_Se_3_ phase [31,32], whereas the peak at 252 cm^−1^ is consistent with the Sb_2_O_3_ phase. As this second phase was not observed in the XRD pattern and XPS spectra, the small Raman peak can be attributed to oxidation of the Sb_2_Se_3_ surface to Sb_2_O_3_ by the high-density laser (1 mW/μm^2^) of the Raman II instrument [31].

### 3.2. Crystallin Structure and Morphology of β-Cu_2_Se Nanowires

**Figure 2:**
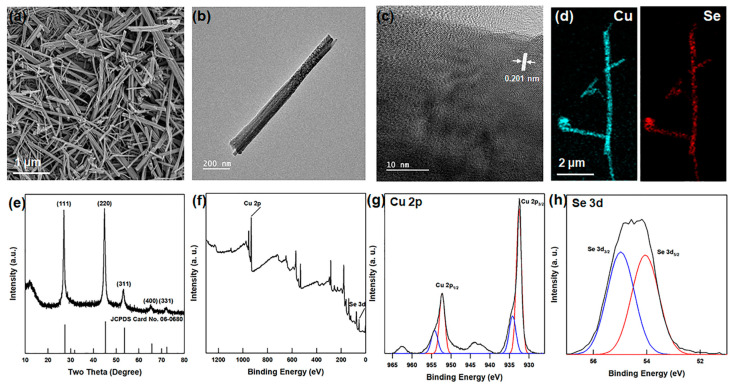
(**a**) FE-SEM image (**b**) FE-TEM (**c**) high-resolution of FE-TEM images of β-Cu_2_Se nanowires. EDS elemental mapping of (**d**) Cu and Se in β-Cu_2_Se nanowires. (**e**) XRD patterns and (**f**) XPS survey spectrum of β-Cu_2_Se nanowires. High-resolution XPS spectra of (**g**) Cu 2p peaks and (**h**) Se 3d peaks in β-Cu_2_Se nanowires.

The β-Cu_2_Se nanowires with diameters of 100−200 nm diameter and lengths of 1–2 μm were obtained as shown in Figure 2a.

Further, the FE-TEM images in Figure 2b,c exhibit 0.201 nm lattice fringes corresponding to the (2 2 0) crystal plane. In addition, the EDS mapping images in Figure 2d,e indicate an atomic ratio of Cu:Se = 68.14:31.86, which is close to the stoichiometric ratio. Meanwhile, the XRD pattern of β-Cu_2_Se in Figure 2f exhibits a diffraction peak with a lattice constant of a = 0.5692 nm, corresponding to the cubic structure (JCPDS #06-0680, a = 0.5759 nm). The absence of any peaks of the second phases in the XRD pattern corresponding to other phases indicates the high purity of the β-Cu_2_Se nanowires.

For further characterization, the XPS spectra of the β-Cu_2_Se nanowires are presented in Figure 2g,i. Here, the presence of the Cu^+^ oxidation state is indicated by the Cu 2p_3/2_ and Cu 2p_1/2_ peaks located at binding energies of 933.66 and 954.56 eV, respectively (Figure 2h). The relatively weaker peaks at 932.38 and 952.33 eV indicate the presence of the Cu^2+^ oxidation state, but other phases such as CuO are not observed. In addition, the Se 3d5/2 and Se 3d3/2 peaks are located at binding energies of 54.01 and 54.94 eV, respectively (Figure 2i). Further, the atomic ratio of Cu:Se is seen to be 64.76:35.24, which is in agreement with that obtained from the EDS mapping ratio and with the stoichiometric ratio. The Raman spectrum of the β-Cu_2_Se nanowire is provided in Figure S2. Here, the peak at 260 cm^−1^ corresponds to the previously reported Raman data for β-Cu_2_Se [33]. In contrast to the Raman spectrum of the Sb_2_Se_3_ nanowires (Figure S1), no oxidized peak is observed for the β-Cu_2_Se nanowires. This confirms the high purity of the synthesized β-Cu_2_Se nanowires.

### 3.3. Confirmation of PANI Coated Sb_2_Se_3_/β-Cu_2_Se Nanowire Powders

**Figure 3:**
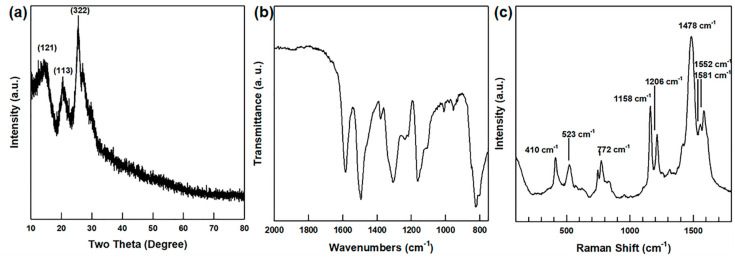
(**a**) XRD patterns, (**b**) FT-IR spectrum, and (**c**) Raman spectrum of de-doped PANI powder.

The XRD pattern of the de-doped PANI is presented in Figure 3a and is in agreement with the previously reported data [34].

In addition, the FT-IR spectrum of the de-doped PANI is presented in Figure 3b. Here, the peaks at 1587 and 1490 cm^−1^ are attributed to the C=C stretching vibrations of the quinoid and benzenoid ring, respectively; the peaks at 1300 and 1240 cm^−1^ indicate the C-N stretching of the benzenoid ring, and the peak at 1151 cm^−1^ indicates the N=quinoid-ring=N vibrational mode [35]. Taken together, the FT-IR and XRD results demonstrate the successful synthesis of the de-doped PANI with the emeraldine structure incorporating both the benzenoid and quinoid rings.

**Figure 4:**
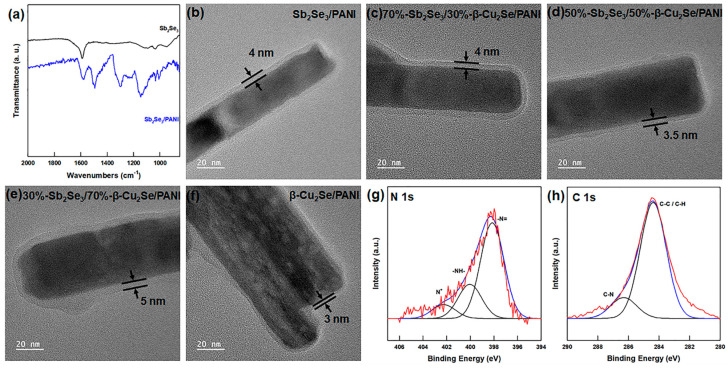
(**a**) FT-IR spectrum of Sb_2_Se_3_ nanowires and Sb_2_Se_3_/PANI powder. High-resolution FE-TEM images of (**b**) Sb_2_Se_3_/PANI, (**c**) 70%-Sb_2_Se_3_/30%-β-Cu_2_Se/PANI, (**d**) 50%-Sb_2_Se_3_/50%-β-Cu_2_Se/PANI (**e**), 30%-Sb_2_Se_3_/70%-β-Cu_2_Se/PANI, and (**f**) β-Cu_2_Se/PANI powders. High-resolution XPS spectra of (**g**) N 1s peaks and (**h**) C 1s peaks in 70%-Sb_2_Se_3_/30%-β-Cu_2_Se/PANI powders.

By comparison, the FT-IR spectrum of the composite Sb_2_Se_3_/β-Cu_2_Se/PANI material in Figure 4a reveals the appearance of peaks at 1591, 1490, 1300, 1240, and 1151 cm^−1^ due to the PANI coated on the surface of Sb_2_Se_3_ and β-Cu_2_Se nanowires.

Further, the FE-SEM images of the composite materials with various ratios of Sb_2_Se_3_ and β-Cu_2_Se nanowires in Figure S3 reveal the change in morphology and increased roughness of the Sb_2_Se_3_/β-Cu_2_Se/PANI nanowire surface. In addition, the FE-TEM images in Figure 4b–f indicate that the PANI is coated on the surface of the Sb_2_Se_3_/β-Cu_2_Se nanowires with a uniform thickness of 4−5 nm. For further characterization, the N 1s and C 1s peaks in the XPS spectra of the 70%-Sb_2_Se_3_/30%-β-Cu_2_Se/PANI composites are presented in Figure 4g,h. Here, the peaks at 398.15, 400.04, and 402.25 eV are respectively attributed to the −N= bonds, the −NH− bonds, and the N^+^ species of the emeraldine base PANI [36]. Meanwhile, the binding energies of 284.38 and 286.31 eV are attributed to the C−C/C−H bonds and the C−N bonds, respectively, of the emeraldine based PANI [36]. Taken together, the FE-TEM and XPS results demonstrate the successful formation of PANI on the surface of the nanowires.

### 3.4. Thermoelectric Properties of Sb_2_Se_3_/β-Cu_2_Se/PANI Flexible Films and TEG Properties

**Figure 5:**
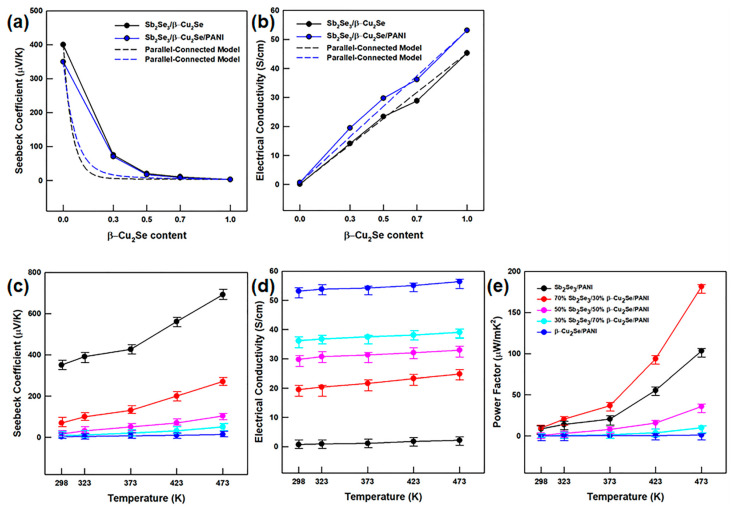
β-Cu_2_Se-ratio-dependent (**a**) Seebeck coefficients and (**b**) electrical conductivities of various ratios of Sb_2_Se_3_/β-Cu_2_Se/PANI films at room temperature. Temperature-dependent (**c**) Seebeck coefficients, (**d**) electrical conductivities, and (**e**) power factors of various ratios of Sb_2_Se_3_/β-Cu_2_Se/PANI films.

The Sb_2_Se_3_/β-Cu_2_Se/PANI films were synthesized from the Sb_2_Se_3_/β-Cu_2_Se/PANI powders with various ratios of Sb_2_Se_3_ and β-Cu_2_Se as described in Section 2.2.3. The flexible properties of the obtained films are indicated in Figure S4, while the Seebeck coefficients (S) and electrical conductivities (σ) of the Sb_2_Se_3_/β-Cu_2_Se nanowires and Sb_2_Se_3_/β-Cu_2_Se/PANI films are indicated in Figure 5a,b.

Here, the pure Sb_2_Se_3_ and β-Cu_2_Se exhibit Seebeck coefficients of 400 and 3−4 μV/K, respectively, while the Seebeck coefficient of the Sb_2_Se_3_/β-Cu_2_Se composite is seen to decrease with increasing proportion of β-Cu_2_Se. Meanwhile, the electrical conductivity of the Sb_2_Se_3_/β-Cu_2_Se composites is seen to increase with the increasing proportion of β-Cu_2_Se due to the high electrical conductivity of β-Cu_2_Se (45.3 S/cm^−1^). Compared to the non-coated Sb_2_Se_3_/β-Cu_2_Se composite, the Sb_2_Se_3_/β-Cu_2_Se/PANI film exhibits a lower Seebeck coefficient and a higher electrical conductivity due to the high electrical conductivity of PANI (i.e., 360 S/cm) [16]. These trends in the Seebeck coefficient and electrical conductivity can be explained by a parallel-connected model, as described in the Supporting Information. Although the complicated interfacial interactions can distort the electrical conductivity curves and, thus, lead to inaccuracy, the parallel-connected model can be considered a useful guideline [37,38,39]. The results of the parallel-connected model are indicated by the dashed line in Figure 5a,b, while the Seebeck coefficients, electrical conductivities, and power factors of the Sb_2_Se_3_/β-Cu_2_Se nanowires and Sb_2_Se_3_/β-Cu_2_Se/PANI films over the temperature range of room temperature to 473 K are presented in Figure 5c−e.

In all cases, the Seebeck coefficients and electrical conductivities are seen to increase with the increasing temperature. In addition, the maximum power factor (*PF*), as calculated using Equation (2), is 181.61 μW/m·K^2^ for the 70%-Sb_2_Se_3_/30%-β-Cu_2_Se/PANI film.

**Figure 6:**
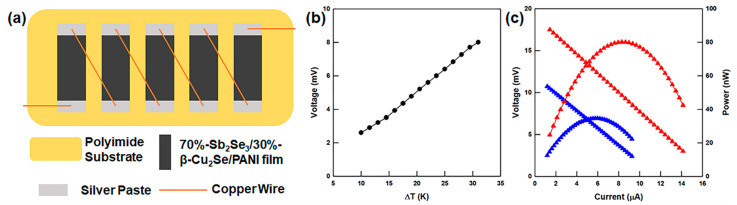
(**a**) Schematic diagram of the TEG structure, (**b**) open-circuit voltage at various temperature differences, and (**c**) output voltage and output power versus current at temperature differences at 20 K and 30 K.

The flexible film of thermoelectric materials can be used as a TEG for wearable or portable devices. Hence, the highest-performing material in the present study, namely the 70%-Sb_2_Se_3_/30%-β-Cu_2_Se/PANI film, was used to fabricate a TEG, as shown in Figure 6a. The open-circuit voltage (*V_oc_*) and output power of the device are indicated in Figure 6b,c. The open-circuit voltage of the fabricated TEG was measured under a temperature difference of Δ*T* = 30 K and reached a value of 7.9 mV. The theoretical value of the open-circuit voltage was calculated using Equation (3):

(3) $$V_{oc}=N\cdot \left|S\right|\cdot \Delta T$$

where *N* is the number of TEG legs [39].

The output power (*P*) was calculated using Equation (4):

(4) $$P=I^{2}\cdot R_{load}={\left(\frac{V_{oc}}{R_{in}+R_{load}}\right)}^{2}\cdot R_{load}$$

where *I*, *R_load_*, and *R_in_* are respectively the output current, the load resistance, and the internal resistance of the homemade TEG^37^. The *R_in_* and *R_load_* values were both 770 Ω, and the calculated maximum output power was 80.1 nW at Δ*T* = 30 K.

## 4. Conclusions

Two nanowire materials, namely Sb_2_Se_3_ and β-Cu_2_Se, were synthesized via hydrothermal reaction and water evaporation-induced self-assembly methods, respectively. The conducting polymer, PANI was then formed on the Sb_2_Se_3_ and β-Cu_2_Se nanowire surfaces in order to improve their electrical conductivities. Composite PANI-coated materials with various ratios of Sb_2_Se_3_ and β-Cu_2_Se were produced, and their thermoelectric properties were measured. The 70%-Sb_2_Se_3_/30%-β-Cu_2_Se/PANI film was shown to provide the highest power factor of 181.61 μW/m·K^2^ at 473 K. In addition, a thermoelectric generator was fabricated from five legs of the 70% Sb_2_Se_3_/30% β-Cu_2_Se/PANI film and was found to provide an open-circuit voltage of 7.9 mV and an output power of 80.1 nW at ΔT = 30 K. This study demonstrates that the fabricated flexible TEG which combines the high performance of inorganic thermoelectric materials with flexibility of a polymer has potential application as a next-generation power generator for wearable or portable devices. In addition, this study can also influence other electronic devices requiring compact power generators.

## Data Availability

The data presented in this study are available on the request from the corresponding author.

## Supplementary Materials

The following are available online at https://www.mdpi.com/article/10.3390/polym13091518/s1, Figure S1: Raman spectrum of Sb_2_Se_3_ nanowires, Figure S2: Raman spectrum of β-Cu_2_Se nanowires, Figure S3: FE-SEM images of Sb_2_Se_3_/β-Cu_2_Se nanowires before and after PANI coating on the nanowire surfaces, Figure S4: Raman spectrum of 70%-Sb_2_Se_3_/30%-β-Cu_2_Se/PANI powders, Figure S5: Macro-scaled morphology of the 70%-Sb_2_Se_3_/30%-β-Cu_2_Se/PANI flexible film.
