# Supplementary material for: Thermoelectric Generator Using Polyaniline-Coated Sb2Se3/β-Cu2Se Flexible Thermoelectric Films
Source: Polymers (Basel). 2021 May 9;13(9):1518. doi: 10.3390/polym13091518 (PMC8125897; doi:10.3390/polym13091518)
Supplement: Supplementary file 1 [file polymers-13-01518-s001.zip › polymers-1198915-supplementary.pdf]

polymers

MDPI

Supplementary Information: Thermoelectric generator using polyaniline-coated Sb_2Se_3/β-Cu_2Se flexible thermoelectric films

Minsu Kim, Dabin Park and Jooheon Kim

1. Figures

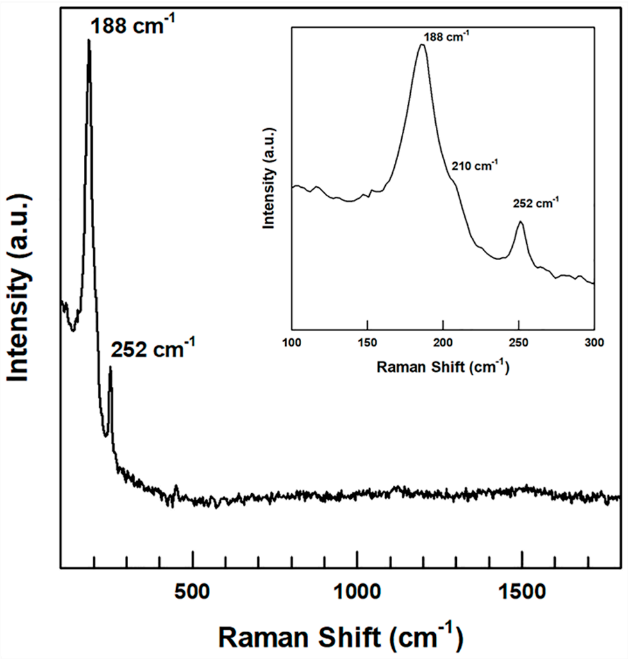

Figure S1. Raman spectrum of Sb_2Se_3 nanowires.

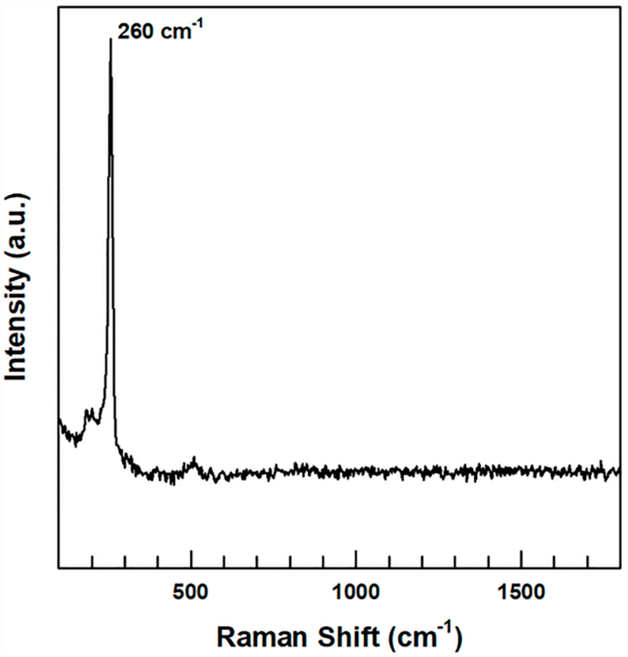

Figure S2. Raman spectrum of β-Cu_2Se nanowires.

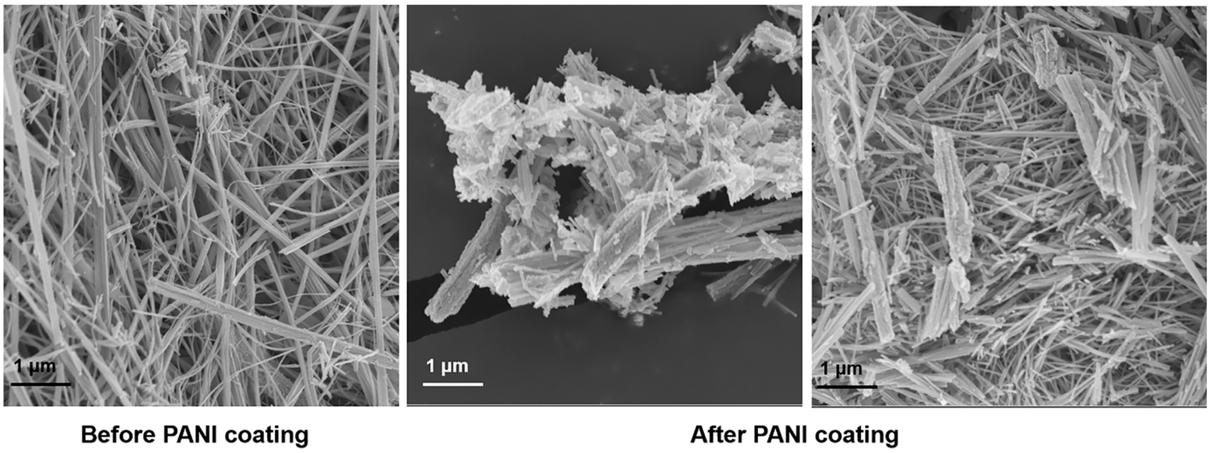

Figure S3. FE-SEM images of Sb_2Se_3/β-Cu_2Se nanowires before and after PANI coating on the nanowire surfaces.

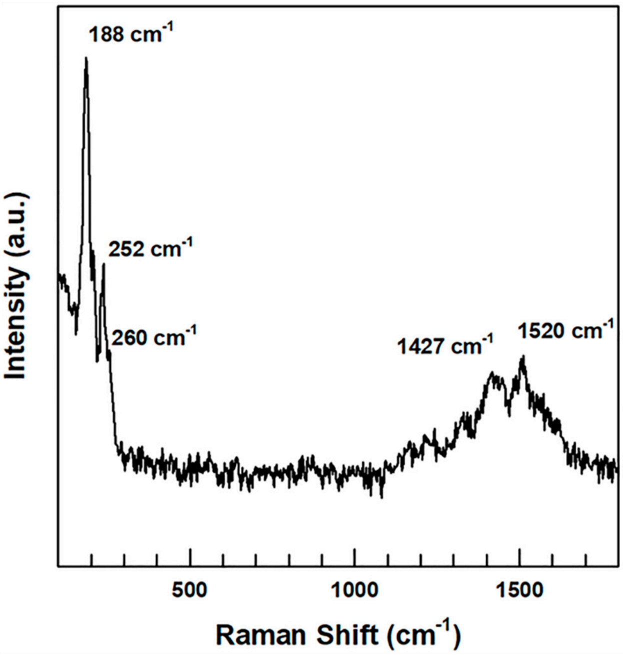

Figure S4. Raman spectrum of 70%-Sb_2Se_3/30%-β-Cu_2Se/PANI powders.

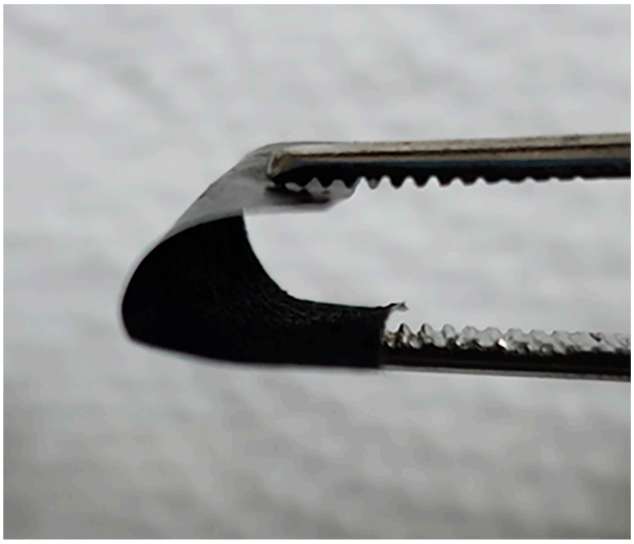

Figure S5. Macro-scaled morphology of the 70%-Sb_2Se_3/30%-β-Cu_2Se/PANI flexible film.

2. Supporting Information

1.1. Parallel-connected model

Detailed description for the parallel-connected model for the Sb_2Se_3/β-Cu_2Se film and Sb_2Se_3/β-Cu_2Se/PANI film with different content of β-Cu_2Se nanowires. The parallel-connected model for the electrical conductivity and Seebeck coefficient of the Sb_2Se_3/β-Cu_2Se and Sb_2Se_3/β-Cu_2Se/PANI can be written as:

$$\sigma_{C,P} = (1 - x_T)\sigma_P + x_T\sigma_T \tag{1}$$

$$S_{C,P} = \frac{(1 - x_T)\sigma_P S_P + x_T\sigma_T S_T}{(1 - x_T)\sigma_P + x_T\sigma_T} \tag{2}$$

where $\sigma_{C,P}$, $S_{C,P}$, x_T, σ_P, σ_T, S_P, and S_T are the parallel-connected electrical conductivity and Seebeck coefficient of the Sb_2Se_3/β-Cu_2Se, volume fraction of the β-Cu_2Se nanowires, electrical conductivity of the Sb_2Se_3 nanowires, electrical conductivity of the β-Cu_2Se nanowires, Seebeck coefficients of the Sb_2Se_3 nanowires, and Seebeck coefficient of the β-Cu_2Se nanowires, respectively [1,2].

References

1. D. Park, M. Kim, J. Kim, Conductive PEDOT: PSS-Based Organic/Inorganic Flexible Thermoelectric Films and Power Generators, Polymers, 13(2) (2021).
2. H. Ju, D. Park, J. Kim, Thermoelectric enhancement in multilayer thin-films of tin chalcogenide nanosheets/conductive polymers, Nanoscale, 11(34) (2019), 16114-16121.
